# Influence of Mo and Ni Alloying on Recrystallization Kinetics and Phase Transformation in Quenched and Tempered Thick Steel Plates

**DOI:** 10.3390/ma19020290

**Published:** 2026-01-10

**Authors:** Xabier Azpeitia, Unai Mayo, Nerea Isasti, Eric Detemple, Hardy Mohrbacher, Pello Uranga

**Affiliations:** 1 Ceit-Basque Research & Technology Alliance (BRTA), 20018 Donostia-San Sebastián, Basque Country, Spain; xazpeitia@ceit.es (X.A.); nisasti@ceit.es (N.I.); puranga@ceit.es (P.U.); 2 Mechanical and Materials Engineering Department, University of Navarra-Tecnun, 20018 Donostia-San Sebastián, Basque Country, Spain; 3 Aktien-Gesellschaft der Dillinger Hüttenwerke, 66763 Dillingen/Saar, Germany; eric.detemple@dillinger.biz; 4 NiobelCon BV, 2970 Schilde, Belgium; hm@niobelcon.net; 5 Department of Materials Engineering (MTM), KU Leuven, 3001 Leuven, Belgium

**Keywords:** martensite, thermomechanical simulations, CCT diagrams, molybdenum, nickel

## Abstract

The production of heavy gauge quenched and tempered steel plates requires alloying strategies that ensure adequate hardenability and microstructural uniformity under limited cooling rates. Molybdenum (Mo) and nickel (Ni) are key elements in this context, as they influence both hot-working behavior and phase transformation kinetics. This study investigates the effect of Mo (0.25–0.50 wt%) and Ni (0–1.00 wt%) additions on static recrystallization and transformation behavior using laboratory thermomechanical simulations representative of thick plate rolling conditions. Multipass and double-hit torsion tests were performed to determine the non-recrystallization temperature (*Tnr*) and quantify softening kinetics, while dilatometry was employed to construct Continuous Cooling Transformation (CCT) diagrams and assess hardenability. Results indicate that Mo significantly increases *Tnr* and delays recrystallization through a solute drag mechanism, whereas Ni exerts a minor but measurable effect, likely associated with stacking fault energy rather than classical solute drag. Both elements reduce ferrite and bainite transformation temperatures, enhancing hardenability; however, Mo alone cannot suppress ferrite formation at practical cooling rates, requiring combined Mo–Ni additions to achieve fully martensitic microstructures. These findings provide insight into alloy design for thick plate applications and highlight the limitations of existing predictive models for Ni-containing steels.

## 1. Introduction

The production of heavy gauge high-strength quenched and tempered plates relies heavily on the strategic addition of alloying elements like molybdenum and nickel. These elements are essential for enhancing the mechanical properties, corrosion resistance, and weldability of the steel plates, making them suitable for demanding applications in industries such as construction, shipbuilding, and heavy lifting equipment. The ability of Mo and Ni to improve the hardenability of the steel during quenching is particularly critical, as it allows for the formation of a fine-grained martensitic microstructure that offers superior strength and toughness [1,2].

In the conventional route, slabs are reheated (~1100–1225 °C), rough-rolled in the full-recrystallization regime, and air-cooled to room temperature; the plate is then re-austenitized (typically 880–930 °C, grade-dependent), water-quenched, and tempered (≈500–650 °C) to achieve tempered martensite with the required toughness and weldability [3,4]. Re-austenitization homogenizes the prior-austenite into equiaxed grains before quenching, but centerline cooling is often limited in heavy gauges, leading to bainite–martensite mixtures unless hardenability is sufficiently high [5,6]. This can lead to the formation of undesired softer constituents such as ferrite and bainite within the martensitic matrix, potentially compromising the overall mechanical performance of the material. Additionally, during hot working, the behavior of Mo and Ni significantly influences the microstructural evolution and phase transformations, further affecting the final properties of the plates [7,8,9].

Mo plays a crucial role in delaying recrystallization and inhibiting grain growth during hot working, primarily due to the drag effect of Mo atoms, which impede dislocation movement and grain boundary migration [10]. Ni, on the other hand, impacts the kinetics of recrystallization and grain growth by promoting a higher recrystallization temperature and reducing the recrystallization rate, a phenomenon linked to the increase in stacking fault energy rather than solute drag [11].

In addition to the detrimental effect of thermal gradients along the thickness, the production of thick plates is particularly challenging when lower reductions are applied during the rolling process. As the strain penetration is limited, especially towards the centerline of the material, it is essential to define rolling passes that promote microstructural homogeneity throughout thickness. An inadequate austenite conditioning increases the risk of forming heterogeneous martensitic microstructures with softer bainitic constituents, which in turn can reduce toughness [12]. Thus, understanding the static recrystallization kinetics and determining the non-recrystallization temperature (*Tnr*) for deformation conditions similar to thick plate rolling, characterized by low per-pass reductions and relatively long interpass times, are crucial for optimizing rolling schedules.

This study provides a comprehensive analysis of the hot-working behavior and phase transformation kinetics in quenched and tempered thick plates, focusing on the influence of Mo (0.25–0.50 wt%) and Ni (0–1.00 wt%) alloying under deformation conditions representative of industrial rolling schedules. Laboratory thermomechanical simulations were employed to replicate the low strain per pass and extended interpass times typical of thick plate rolling. Multipass torsion tests were used to determine the non-recrystallization temperature (*Tnr*) and quantify static recrystallization kinetics, while dilatometry enabled the construction of Continuous Cooling Transformation (CCT) diagrams to assess hardenability. Unlike previous studies that examine Mo or Ni effects individually, this work delivers a combined evaluation of their interaction on austenite conditioning and transformation behavior. Furthermore, the results reveal limitations of existing recrystallization models when applied to Ni-containing steels, highlighting the need to incorporate stacking fault energy considerations in predictive approaches. The novelty of this work lies in providing the first integrated assessment of Mo–Ni synergy on both recrystallization and phase transformation under thick plate rolling conditions, and in proposing a modified activation energy approach to improve recrystallization modeling for Ni-bearing steels. These findings establish a direct link between alloy design and process parameters, offering guidance for optimizing rolling schedules and heat treatment strategies for heavy-gauge plates.

## 2. Materials and Methods

Table 1 summarizes the chemical composition of the material under analysis. The chemical composition of the steels was determined using Optical Emission Spectroscopy with spark excitation (OES–spark). Five different steels were analyzed, each with varying levels of both Mo and Ni, as well as combinations of these elements. These materials were laboratory-casted in ingots.

**Figure 1:**
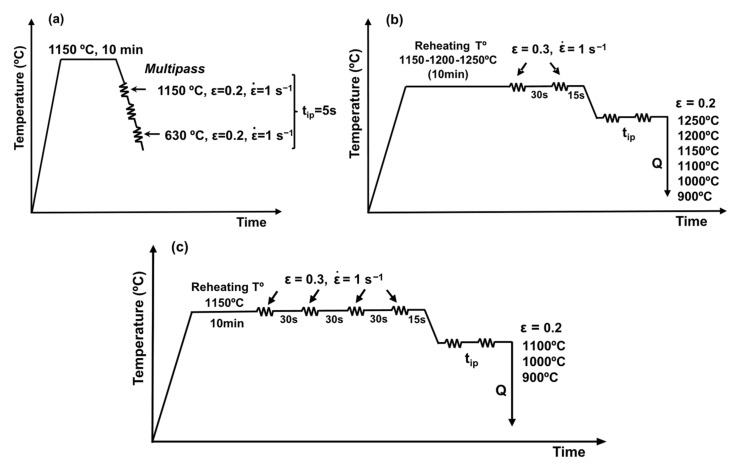
Schematics of the thermomechanical cycles applied in torsion for the definition of (**a**) critical temperatures like *Tnr* and (**b**,**c**) softening kinetics for (**b**) 25Mo, 50Mo, 50Mo-50Ni, 50Mo-100Ni and (**c**) 25Mo-50Ni [9].

To characterize static recrystallization kinetics and identify critical temperatures such as the *Tnr*, a combination of double-hit and multipass torsion tests was employed. Experiments were conducted on a fully automated hot torsion testing system using cylindrical specimens with a gauge length of 15 mm and a diameter of 7.5 mm. Temperature control was achieved via a thermocouple inserted into a drilled hole at the specimen center. The multipass schedule used to determine *Tnr* is depicted in Figure 1a. After an initial austenitization at 1150 °C for 10 min, 27 successive deformation passes were applied with a strain of ε = 0.2 per pass, decreasing the temperature in 20 °C increments from 1150 °C down to 630 °C. A constant strain rate of 1 s^−1^ was maintained throughout the sequence. These tests were conducted on all steels except 50Mo-100Ni; the details are summarized in Table 2.

To investigate static recrystallization behavior, double-hit torsion tests were carried out following the thermomechanical sequences illustrated in Figure 1b,c. Each cycle began with a 10 min reheating stage, after which a refining deformation was applied to produce a uniform, fine-grained austenitic structure prior to the test. The reheating temperature and refinement schedule varied according to alloy design: most steels were reheated at 1150 °C and tested at deformation temperatures below 1100 °C, whereas the 50Mo–50Ni grade required an extended temperature range of 1250, 1200, and 1150 °C, with corresponding adjustments to reheating. In all cases, two preliminary passes of 0.3 strain were introduced to prevent coarse austenite formation, as shown in Figure 1b. For the 25Mo–50Ni steel (Figure 1c), two additional refining passes were necessary to eliminate microstructural heterogeneity. After refinement, specimens were cooled to the selected deformation temperature within the 1250–900 °C interval, and a strain of 0.2 was applied. Fractional softening was determined using the 2% offset criterion, recognized as the most reliable approach for excluding recovery effects when strain-induced precipitation is absent [13]. Table 3 summarizes the experimental conditions for the double hit torsion tests.

**Figure 2:**
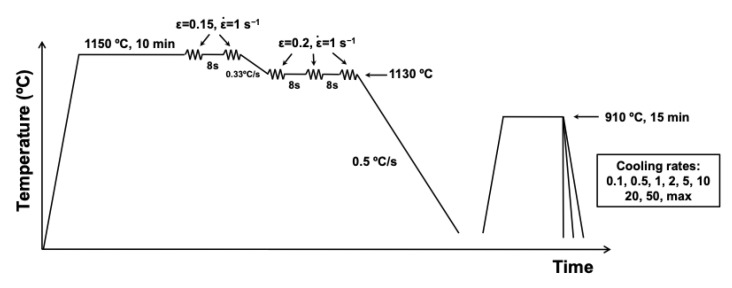
Thermomechanical schedule for the dilatometry tests.

For the construction of CCT diagrams, dilatometry tests were conducted using a Bähr DIL805A/D dilatometer (Bähr-Thermoanalyse GmbH, Hullhorst, Germany). Cylindrical samples of 10 mm length and 5 mm diameter were machined for that purpose. The temperature is measured by welded thermocouple. The thermomechanical schedule applied to the specimens is illustrated in Figure 2. The sequence began with reheating at 1150 °C for 10 min, followed by two deformation passes at 1050 °C with a strain of 0.15 and a strain rate of 1 s^−1^ to produce a refined, fully recrystallized austenitic structure. Subsequently, three additional passes of 0.20 strain were applied at 1130 °C, after which the samples were cooled slowly to room temperature at approximately 0.5 °C/s. A re-austenitization treatment was then performed at 910 °C for 15 min, and controlled cooling was applied at rates ranging from 0.1 °C/s up to maximum quenching intensity to construct the Continuous Cooling Transformation (CCT) diagrams.

For torsion tests, initial grain sizes were measured metallographically in the sub-surface longitudinal section, corresponding to 0.9 of the outer radius of the torsion specimen. The analysis of the austenitic structure was performed using optical microscopy (OM, LEICA DM15000 M, Leica microsystems, Wetzlar, Germany). The specimens were etched by a solution of saturated picric acid and HCl to reveal the austenite grain boundaries in the quenched samples. Austenite grain size distributions were measured considering the mean equivalent diameter method. The measurements were carried out using the QWin v.2.3 image analysis software. For each steel grade, 400–600 austenite grains were measured.

For dilatometry tests, the transformed microstructures were etched in 2% Nital and the austenite prior to transformation was revealed by etching in a solution of saturated picric acid and HCl. Samples were characterized by optical microscopy (OM, LEICA DM15000 M, Leica microsystems, Wetzlar, Germany) and field-emission gun scanning electron microscopy (FEGSEM, JEOL JSM-7100F, Tokyo, Japan). In addition, the austenite grain sizes prior to transformation were measured using the mean equivalent diameter method, with all specimens finally undergoing the Vickers Hardness test, using a 1-kg load.

## 3. Results and Discussion

### 3.1. Definition of the Non-Recrystallization Temperature (Tnr)

The *Tnr* was established through multipass torsion testing for all compositions except the 50Mo-100Ni grade. Stress–strain data obtained from these tests were used to compute the mean flow stress (MFS), defined as the integral of the stress–strain curve normalized by the applied strain per pass. The determination of *Tnr* followed the procedure described by Bai and co-workers [14], which is widely accepted for identifying the transition between complete and partial recrystallization.

**Figure 3:**
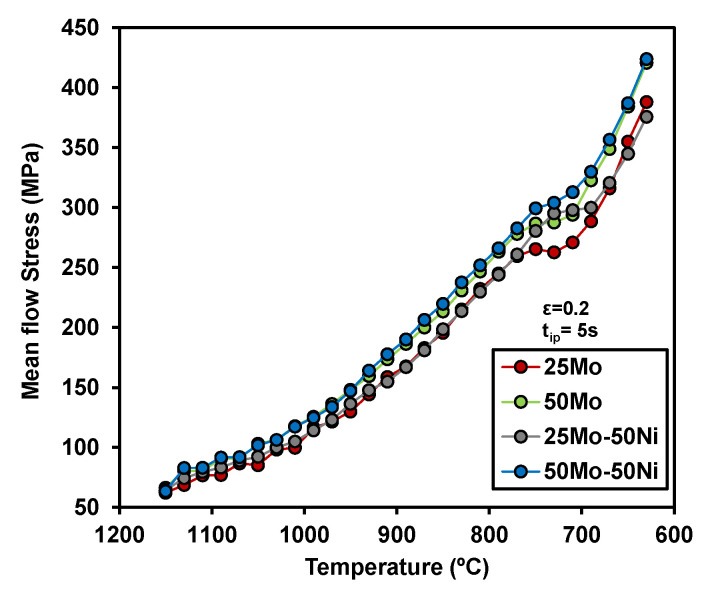
Comparison of the mean flow stress values, derived from multipass torsion tests, as a function of temperature for the different chemical compositions.

Figure 3 shows the effect of chemical composition on the evolution of MFS values for each deformation pass, plotted against the temperature. The lowest MFS values are observed in the 25Mo steel, while the highest values are measured for the 50Mo-50Ni chemistry. This trend reflects the cumulative effect of alloying elements on deformation resistance. Molybdenum and nickel increase the non-recrystallization temperature and retard softening between passes, which promotes strain accumulation. As a result, the stress level rises progressively with each pass, not only due to decreasing temperature but also because incomplete recrystallization leads to work hardening. The term “hardening effect” refers to this progressive increase in flow stress caused by solute drag and recovery inhibition, rather than precipitation strengthening, since strain-induced precipitation was absent under the applied conditions. Additionally, a noticeable decrease in MFS values is observed between deformation passes at 750 °C and 710 °C, which is associated with the onset of the transformation from austenite to ferrite.

**Figure 4:**
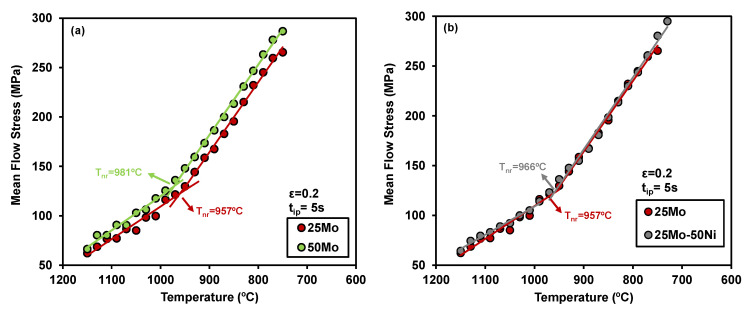
Mean flow stress derived from multipass torsion tests as a function of temperature for (**a**) 25Mo vs. 50Mo and (**b**) 25Mo vs. 25Mo-50Ni steels. *Tnr* values are also incorporated.

Figure 4 compares the MFS values for steels with different Mo and Ni contents, illustrating how these alloying elements affect MFS behavior. Two distinct deformation regimes can be observed. At higher temperatures, full recrystallization occurs between passes, and the increase in stress from pass to pass is primarily attributed to the temperature decrease. Conversely, at lower austenite temperatures, strain accumulation becomes evident due to partial recrystallization [15]. Following the standard approach [14], *Tnr* was identified as the intersection of the linear regressions fitted to the data in each regime. The *Tnr* values obtained were 957 °C, 981 °C, 966 °C, and 980 °C for the 25Mo, 50Mo, 25Mo-50Ni, and 50Mo-50Ni compositions, respectively.

A significant increase in *Tnr* is observed with a higher Mo content (as shown in Figure 4a), indicating the ability of Mo to hinder dislocation movement and grain boundary migration [10]. By contrast, the impact of Ni on *Tnr* appears less pronounced and the combination of both microalloying elements does not produce a greater effect compared to Mo alone (*Tnr* values for the 50Mo and 50Mo-50Ni compositions are nearly identical). Only a slight increase in *Tnr* is seen when comparing the 25Mo and 25Mo-50Ni chemistries (see Figure 4b).

From the preceding discussion, it appears that the dominant factor affecting the *Tnr* is the solute drag phenomenon. Molybdenum, due to its considerably larger atomic radius compared to iron, tends to segregate at austenite grain boundaries [16,17]. The efficiency of solute drag increases when the alloying element exhibits a pronounced atomic size mismatch with the matrix atoms (Fe), as this enhances lattice distortion and consequently strengthens the drag force [18]. In this regard, molybdenum demonstrates a stronger effect than nickel, which has an atomic size closer to that of iron. Furthermore, Mo possesses a relatively low self-diffusion coefficient, which limits the mobility of segregated atoms and makes their removal from grain boundaries more difficult, thereby amplifying the drag effect [19].

The comparatively minor effect of Ni on the *Tnr* can be explained by its metallurgical characteristics. Unlike Mo, which exhibits a strong solute drag effect due to its larger atomic radius and low self-diffusion coefficient, Ni has an atomic size close to that of Fe and diffuses more rapidly, reducing its ability to impede grain boundary migration. Consequently, Ni does not significantly increase *Tnr* through solute drag. Instead, its influence is primarily associated with the SFE. Higher SFE facilitates dynamic recovery by promoting cross-slip, which lowers stored energy and decreases the driving force for nucleation. This mechanism explains why Ni additions can slightly delay recrystallization kinetics without markedly altering *Tnr*, as observed in this study. These findings are consistent with previous reports attributing the effect of Ni to recovery-related phenomena rather than classical solute drag [11].

### 3.2. Analysis of the Static Recrystallization Kinetics

To investigate the static recrystallization kinetics, double-pass torsion tests were conducted (see the thermomechanical cycles depicted in Figure 1b,c). As mentioned before, the reheating temperature and refining step varied depending on the alloy composition. For 25Mo, 50Mo and 50Mo-100Ni steels, a reheating temperature of 1150 °C and deformation temperatures below 1100 °C were applied following the thermomechanical cycle depicted in Figure 1b. In contrast, for the 50Mo-50Ni steel, the reheating temperature was increased to 1250 °C and 1200 °C, as the deformation temperature range was extended for 1250 °C, 1200 °C and 1150 °C (Figure 1b). Finally, for 25Mo-50Ni steel, the thermomechanical cycle shown in Figure 1c was employed, as additional refining deformation passes were needed in order to avoid austenite heterogeneity. Despite the variations in reheating temperatures and austenite conditioning, the refining step resulted in a consistent austenite grain structure across all tested cases. Mean austenite sizes of 44 µm, 36 µm, 30 µm, 42 µm and 38 µm are measured for 25Mo, 50Mo, 25Mo-50Ni, 50Mo-50Ni and 50Mo-100Ni chemistries, respectively.

**Figure 5:**
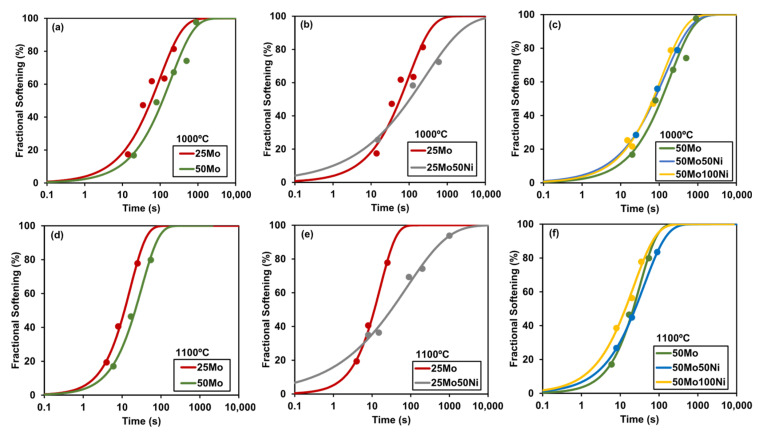
Static recrystallization curves for double-hit tests by hot torsion: (**a**–**c**) 1000 °C and (**d**–**f**) 1100 °C deformation temperatures with a reheating temperature of 1150 °C.

In Figure 5, the impact of deformation temperature and chemical composition on fractional softening curves can be evaluated. To examine the effects of varying Mo and Ni additions, three comparison groups are defined. Figure 5a,d illustrate the impact of Mo additions at deformation temperatures of 1000 °C and 1100 °C, respectively. The addition of Mo clearly delays static recrystallization kinetics at both temperatures.

In Figure 5b,c,e,f, the effect of different Ni additions (from 0 to 1% Ni) is shown. For high Mo chemistries (see Figure 5c,f), Ni additions have no observable effect across the analyzed deformation temperatures. However, in low Mo steels (see Figure 5b,e), a delay in static recrystallization kinetics is observed due to a reduction in the Avrami curve *n*-value. The underlying mechanism driving these changes remains unclear, as the observations are not systematic. As suggested in [2], Ni may enhance restoration kinetics in austenite due to its higher stacking fault energy (SFE), potentially delaying recrystallization kinetics. The observed decrease in Avrami *n*-value with Ni addition aligns with findings by Ji et al. [20] for Fe–30 wt % Ni model alloys, indicating that Ni reduces nucleation density and fosters growth-controlled recrystallization through enhanced dynamic recovery, consistent with its effect on stacking fault energy.

The time required for 50% recrystallization, denoted as *t*_0.5_, is influenced by several factors, including the initial austenite grain size (*D*_0_), deformation parameters (temperature, strain, and strain rate), and the amount of microalloying elements in solid solution. Numerous empirical models for estimating *t*_0.5_ have been reported in the literature. In this work, the equation proposed by Fernandez et al. [21] was adopted as the starting point, incorporating the solute drag effect described by Jonas [22].

(1) $$t_{0.5}=9.92\times 10^{-11}\cdot D_{0\;}\cdot \varepsilon^{{-5.6D_{0}}^{-0.15}}\cdot {\dot{\varepsilon }}^{-0.53}\cdot exp\left(\frac{180,000}{RT}\right)\cdot exp\left[\left(\frac{275,000}{T}-185\right)\cdot \left[Nb+0.0936Mo\right]\right]$$

**Figure 6:**
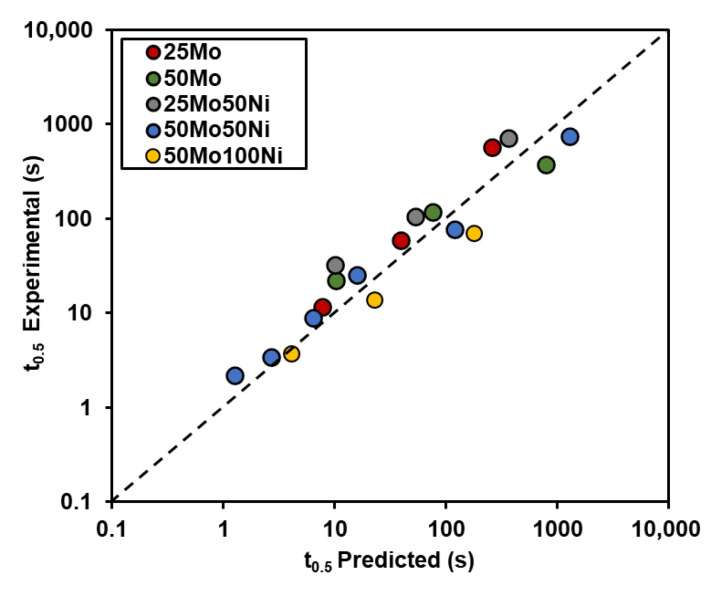
Comparison between experimental and predicted *t*_0.5_ values considering the effect of Ni in the activation energy for recrystallization in Equation (1).

Equation (1) provides reasonably accurate predictions for most cases, and previous studies have reported good agreement when applying this approach to steels with high Mo and boron contents [10]. However, for Ni-bearing grades, the predicted values significantly underestimate the experimental results. As noted in [2], nickel may accelerate restoration processes in austenite due to its higher stacking fault energy (SFE), which tends to promote dynamic recovery and reduce the driving force for recrystallization. This mechanism differs from the solute drag effect already considered for Nb and Mo in Equation (1) and can be accounted for by adjusting the activation energy for recrystallization, *Q_rex_*. In its original form in Equation (1), the value for the activation energy for recrystallization is *Q_rex_* = 180,000 J·mol^−1^. Based on the experimental results, the modified expression shown in Equation (2) is proposed, reflecting the increase in activation energy associated with Ni additions. Figure 6 compares the experimentally measured *t*_0.5_ values with those predicted using the modified equation, demonstrating good agreement and reliable predictability for static recrystallization kinetics.

(2) $$Q_{rex}^{*}=180,000+9000\cdot \%Ni\;(J\cdot {mol}^{-1})$$

### 3.3. Influence of Mo and Ni on Phase Transformations

Following the thermomechanical cycle in Figure 2, dilatometry tests simulating a Conventional Quenching (CQ) schedule were carried out for all chemistries. Continuous Cooling Transformation (CCT) diagrams were subsequently generated, and the resulting microstructure and hardness was evaluated to analyze the influence of chemical composition on phase transformation.

The CQ process normalizes the microstructure, resulting in an equiaxed austenite grain morphology across all steels under study. The re-austenitizing conditions applied in this research (910 °C for 15 min) effectively limit significant austenite grain growth, producing relatively fine austenitic structures. Similar initial austenite grain sizes between 14 and 17 µm are obtained across the different steels. It is reported that Mo plays a role in reducing the interface mobility between ferrite and austenite, potentially contributing to grain size reduction at elevated austenite temperatures through the solute drag effect [23]. However, this effect requires sufficient diffusion of Mo to the austenite grain boundaries, which depends on the diffusional range at the given reheat temperature and time. Based on diffusion data for Mo in austenite, the characteristic diffusion distance $L=\sqrt{4Dt},$ where *D* is the diffusion coefficient of Mo in fcc iron and *t* is time. At 910 °C for 15 min, *D* ~ 10^−14^ m^2^/s [24] and *L* is about 6 µm, far smaller than the austenite grain size (14–17 µm). Therefore, under the reheating conditions used in this study, the diffusional range is significantly smaller than the grain size, making the solute drag effect negligible.

**Figure 7:**
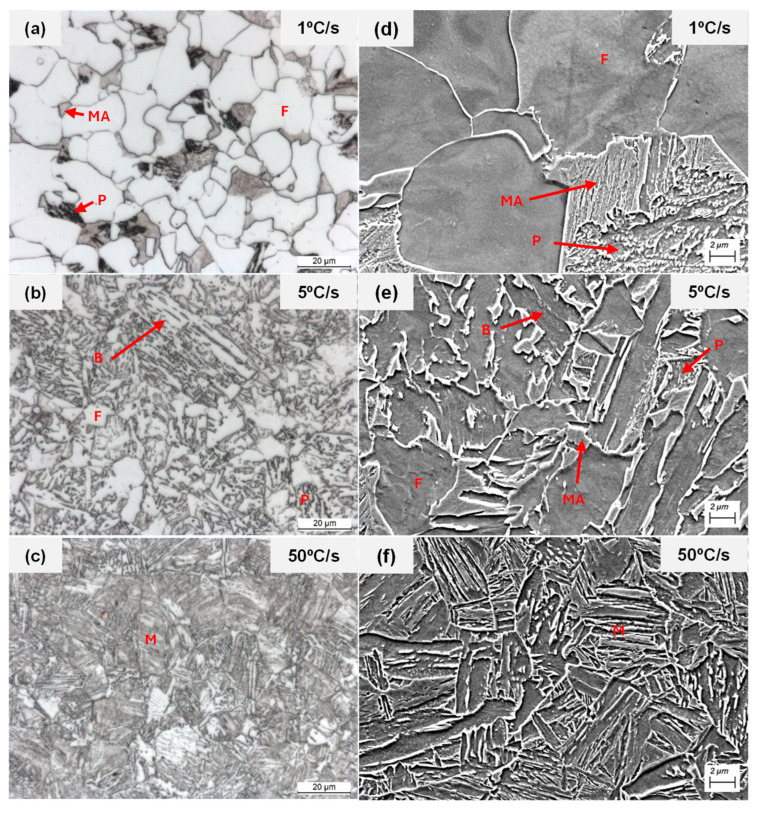
Optical (**a**–**c**) and FEGSEM (**d**–**f**) micrographs of the 25Mo steel for different cooling rates. In the images, F denotes ferrite, P pearlite, B bainite, M martensite, and MA martensite–austenite islands.

**Figure 8:**
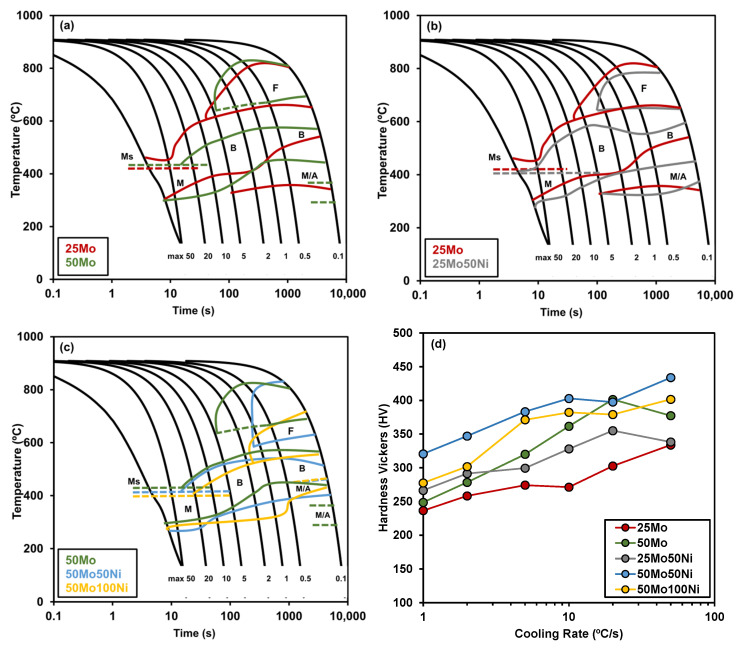
Comparison of CCT diagrams: (**a**) 25Mo and 50Mo; (**b**) 25Mo and 25Mo-50Ni; (**c**) 50Mo, 50No-50Ni and 50Mo-100Ni steels. (**d**) Evolution of Vickers hardness for the different steels and cooling rates. In the charts, F denotes ferrite, B bainite, M martensite, and MA martensite–austenite islands. Ms is the Martensite Start temperature.

Figure 7 presents examples of the microstructures obtained at cooling rates of 1 °C/s, 5 °C/s, and 50 °C/s, for 25Mo steel using optical microscopy and FEGSEM. At a cooling rate of 1 °C/s, the microstructure predominantly consists of polygonal ferrite and pearlite together with isolated Martensite–Austenite (MA) islands (see Figure 7a). At intermediate cooling rates (5 °C/s), polygonal ferrite formation is reduced and a mainly bainitic microstructure is observed. Secondary phases such as MA islands and pearlite are dispersed within the ferrite matrix (see Figure 7e). Finally, at the highest cooling rate of 50 °C/s, the microstructure becomes fully martensitic (Figure 7c,f). This microstructural evolution is generally consistent across all steels. However, the additions of Mo and Ni alter the critical cooling rates necessary to achieve these transformations, leading to variations in the final microstructure. These differences are further corroborated by the changes in Vickers hardness values (see Figure 8d).

Figure 8a–c illustrates the comparison of the CCT diagrams built to examine the effects of varying Mo and Ni additions. A more detailed description and discussion on these, and other steels with similar compositions can be found in Ref. [25]. The impact of increasing Mo content from 0.25 to 0.5% is evaluated in Figure 8a. No clear effect of Mo is noticed regarding the ferritic region. Conversely, the findings indicate that adding Mo tends to delay the bainitic transformation, lowering the corresponding transformation temperatures [1,2]. For steels without Ni (25Mo and 50Mo), martensite formation is restricted when cooling rates exceed 20 °C/s. It should be emphasized that Mo alone cannot completely suppress ferrite formation at practical cooling rates (up to 5 °C/s); only its combination with Ni effectively inhibits ferrite nucleation. The primary contribution of molybdenum to hardenability lies in reducing ferrite nucleation and slowing transformation through a pronounced solute drag effect [25,26].

Similarly, Ni addition to low Mo steel (25Mo) delays bainite transformation (see Figure 8b) but is less effective than Mo enhancing hardenability. This difference is notable at cooling rates above 5 °C/s, as seen in hardness comparisons between 50Mo and 25Mo-50Ni in Figure 8d.

Figure 8c illustrates the influence of increasing Ni content on steel hardenability. The data indicate that Ni addition delays phase transformations, lowering both the start and finish temperatures in the ferritic and bainitic regions. For cooling rates above 2 °C/s, the ferritic region disappears, and achieving a fully martensitic microstructure requires only moderate cooling rates, with martensite formation possible at 5 °C/s. Figure 8a–c also note the experimental *Ms* levels defined from the dilatometry curves. These *Ms* values range from 440 °C for the 50Mo steel down to 400 °C for the 50Mo-100Ni steel [25].

Figure 8d shows that hardness increases with cooling rate for all chemistries, reflecting the suppression of ferrite and bainite and the predominance of martensite at higher cooling intensities. Ni-free steels (25Mo, 50Mo) require substantially higher cooling rates to achieve mostly martensitic structures (onset ≈ 20 °C/s), whereas Mo-Ni grades reach martensite at much lower rates (≈5 °C/s), explaining the higher hardness values recorded across the cooling spectrum for Ni-containing alloys. This trend agrees with dilatometric evidence that faster cooling favors single-stage martensitic transformation and raises macro-hardness, while slower cooling permits diffusional products and mixed microstructures [6,26].

Although Ni generally improves hardenability and lowers transformation temperatures, the hardness response is not strictly monotonic with Ni content. Steels with identical Mo but higher Ni (e.g., 50Mo-50Ni vs. 50Mo-100Ni) do not show proportional hardness gains; in fact, the grade with 1 wt% Ni exhibits slightly lower hardness at high cooling rates. This behavior can be explained by the depression of the martensite start temperature (*Ms*) caused by Ni, which stabilizes austenite and may leave retained austenite even after rapid cooling, reducing overall hardness. Additionally, Ni increases the stacking fault energy (SFE) of austenite, promoting cross-slip and dynamic recovery during prior austenite conditioning, which lowers stored energy and slightly retards martensitic nucleation and growth [2,8,20].

A further observation is the slight decrease in hardness for 25Mo and 25Mo-50Ni at the fastest cooling rate tested. This effect may result from auto-tempering of martensite formed at relatively high temperatures during very rapid cooling. Auto-tempering reduces hardness by early carbide precipitation and carbon redistribution. High-resolution studies and Jominy/dilatometry analyses confirm that hardness can plateau or even drop at extreme quench severities due to microstructural heterogeneity and incomplete martensitic finish [26].

## 4. Conclusions

This study demonstrates that molybdenum and nickel additions exert distinct and complementary effects on the hot-working behavior and phase transformation kinetics of quenched and tempered thick plates. Molybdenum markedly increases the non-recrystallization temperature and delays static recrystallization through a strong solute drag mechanism, whereas nickel shows a minor influence primarily associated with stacking fault energy, without significantly altering *Tnr*. The combined presence of Mo and Ni does not produce a synergistic increase in recrystallization resistance beyond that of Mo alone, confirming Mo as the dominant element for austenite conditioning under rolling conditions characterized by low strain per pass and extended interpass times.

Phase transformation analysis reveals that both elements reduce ferrite and bainite transformation temperatures, improving hardenability. However, Mo alone cannot fully suppress ferrite formation at practical cooling rates, while combined Mo-Ni additions enable martensitic transformation at intermediate cooling rates above 2 °C/s. To achieve fully martensitic microstructures with adequate hardness, Mo and Ni contents should approach 0.5 wt% each. Despite these improvements, conventional quenching of heavy-gauge plates often results in mixed bainitic–martensitic structures at the centerline, highlighting the need for accelerated cooling or optimized alloy design.

From an industrial perspective, rolling schedules for Mo-rich steels should optimize interpass times and apply sufficient strain per pass to promote homogeneous austenite refinement. Heat treatment strategies must consider the residual bainitic fraction in the core, and tempering should be adjusted to balance strength and toughness. These findings provide theoretical guidance for alloy design and process optimization in thick plate production, emphasizing the roles of Mo and Ni in recrystallization inhibition and transformation delay. Furthermore, the observed limitations of current predictive models for Ni-containing steels underline the need to incorporate stacking fault energy effects into future formulations.

## Figures and Tables

**Table 1:** Chemical composition of the selected steels (wt%).

	C	Si	Mn	Al	Mo	Ni	Ti
25Mo	0.14	0.28	1.27	0.018	0.25	0.07	0.033
50Mo	0.15	0.30	1.34	0.016	0.50	0.03	0.025
25Mo-50Ni	0.15	0.30	1.33	0.015	0.25	0.50	0.017
50Mo-50Ni	0.14	0.34	1.30	0.017	0.50	0.50	0.024
50Mo-100Ni	0.17	0.31	1.26	0.026	0.50	1.00	0.026

**Table 2:** Multipass torsion test conditions for *Tnr* definition.

	Reheating Temperature, 1150 °C
Number of passes	27
Strain per pass	0.2
Strain Rate (s^−1^)	1
Interpass time (s)	5
First pass T (°C)	1150
Last pass T (°C)	630

**Table 3:** Experimental conditions for double-hit torsion tests.

Steel	Double-Pass Torsion Tests			
	Reheating T (°C)	Deformation T (°C)	Strain per Pass	Strain Rate (s^−1^)
25Mo	1150	1100	0.2	1
	1150	1000	0.2	1
	1150	900	0.2	1
50Mo	1150	1100	0.2	1
	1150	1000	0.2	1
	1150	900	0.2	1
25Mo-50Ni	1150	1100	0.2	1
	1150	1000	0.2	1
	1150	900	0.2	1
50Mo-50Ni	1250	1250	0.2	1
	1200	1200	0.2	1
	1150	1150	0.2	1
	1150	1100	0.2	1
	1150	1000	0.2	1
	1150	900	0.2	1
50Mo-100Ni	1200	1200	0.2	1
	1150	1100	0.2	1
	1150	1000	0.2	1

## Data Availability

The original contributions presented in this study are included in the article. Further inquiries can be directed to the corresponding author.
